# Haptic based fundamentals of laparoscopic surgery simulation for training with objective assessments

**DOI:** 10.3389/frobt.2024.1363952

**Published:** 2024-05-30

**Authors:** P. Abinaya, M. Manivannan

**Affiliations:** Haptics Laboratory, Department of Applied Mechanics and Biomedical Engineering, Indian Institute of Technology Madras, Tamil Nadu, India

**Keywords:** laparoscopic training simulator, FLS task, haptics, virtual reality, laparoscopic tool force model, and psychophysics

## Abstract

Force is crucial for learning psychomotor skills in laparoscopic tissue manipulation. Fundamental laparoscopic surgery (FLS), on the other hand, only measures time and position accuracy. FLS is a commonly used training program for basic laparoscopic training through part tasks. The FLS is employed in most of the laparoscopic training systems, including box trainers and virtual reality (VR) simulators. However, many laparoscopic VR simulators lack force feedback and measure tissue damage solely through visual feedback based on virtual collisions. Few VR simulators that provide force feedback have subjective force metrics. To provide an objective force assessment for haptic skills training in the VR simulators, we extend the FLS part tasks to haptic-based FLS (HFLS), focusing on controlled force exertion. We interface the simulated HFLS part tasks with a customized bi-manual haptic simulator that offers five degrees of freedom (DOF) for force feedback. The proposed tasks are evaluated through face and content validity among laparoscopic surgeons of varying experience levels. The results show that trainees perform better in HFLS tasks. The average Likert score observed for face and content validity is greater than 4.6 ± 0.3 and 4 ± 0.5 for all the part tasks, which indicates the acceptance of the simulator among subjects for its appearance and functionality. Face and content validations show the need to improve haptic realism, which is also observed in existing simulators. To enhance the accuracy of force rendering, we incorporated a laparoscopic tool force model into the simulation. We study the effectiveness of the model through a psychophysical study that measures just noticeable difference (JND) for the laparoscopic gripping task. The study reveals an insignificant decrease in gripping-force JND. A simple linear model could be sufficient for gripper force feedback, and a non-linear LapTool force model does not affect the force perception for the force range of 0.5–2.5 N. Further study is required to understand the usability of the force model in laparoscopic training at a higher force range. Additionally, the construct validity of HFLS will confirm the applicability of the developed simulator to train surgeons with different levels of experience.

## 1 Introduction

Laparoscopy is a type of minimally invasive surgery (MIS) that involves distinct surgical skills compared to open surgery. It has certain limitations, such as diminished depth perception, limited touch feedback, the fulcrum effect, specialized hand-eye coordination, and restricted laparoscopic tool (LapTool) motion (Xin et al., 2006). Hence, MIS needs special training methods such as box trainers, animal models, and virtual reality (VR) simulators (Varras et al., 2020; Hong et al., 2021) to train individual laparoscopic surgical tasks, for example, clipping and cutting, known as part-task training. While the part tasks used in training may differ across simulators, the fundamentals of laparoscopic surgery (FLS), taught by SAGES and the American college of surgeons (ACS), is a widely recognized and commonly used program. (Peters et al., 2004). The FLS Program comprises a didactic curriculum and an assessment tool that focus on fundamental knowledge, clinical judgment, and technical abilities required for basic laparoscopic surgery. The FLS Trainer Box facilitates the assessment of manual technical skills. The FLS evaluation measures movement speed and accuracy for a specific number of task repetitions to assess proficiency.

The FLS consists of five basic tasks: peg transfer, precision cutting, ligating loop, extracorporeal suturing, and intracorporeal suturing. The majority of MIS training simulators incorporate FLS into their curriculum to train on skills such as reduced depth perception, hand-eye coordination, the fulcrum effect, and bi-manual dexterity. It measures the efficiency and precision of the surgeon’s maneuvers while also penalizing any errors specific to the task. However, direct force metrics or assessments of force skills (Xin et al., 2006) are lacking. This is crucial since the LapTool diminishes the touch feedback experienced during MIS. Moreover, the application of excessive force during laparoscopic surgery is a critical contributing factor to tissue injury (Xin et al., 2006). Therefore, there is a significant need for training in force skills. The study discussed in (Horeman et al., 2012) showed improved trainee performance in the needle transfer task on a laparoscopic box trainer due to visual force feedback. A study examined in (Chmarra et al., 2008) compared the box trainer and VR simulator (SIMENDO) without force feedback. The findings demonstrated the importance of force feedback in basic laparoscopic part tasks such as moving balls, rings, and elastic bands using movement metrics. Despite the presence of touch feedback in box trainers and other animal model trainers for actual object interactions, the process of objectively assessing the performance is tough (Varras et al., 2020; Hong et al., 2021). Additionally, manufacturing tissue-like materials remains difficult. Furthermore, it is challenging to simulate real-time anomalies for advanced procedural modules in box trainers.

The VR simulators have the potential to simulate realistic surgical procedures with objective assessments. However, many commercial VR simulators do not provide force feedback while interacting with virtual tissue models, as discussed in (Varras et al., 2020; Hong et al., 2021; Schijven and Jakimowicz, 2003). These simulators measure the skill of tissue manipulation only via the visual feedback of virtual interactions (Hong et al., 2021; Taba et al., 2021). Very few VR simulators provide force feedback for MIS part tasks. However, they use assessment methods such as the objective structured assessment of technical skills (OSATS), the global operative assessment of laparoscopic skills (GOALS), task completion time, motion metrics (distance traveled, path length, velocity, etc.), tissue damage, and task-specific errors. These simulators do not provide any direct objective force-related metrics (Pinzon et al., 2016; Schijven and Jakimowicz, 2003; Lamata et al., 2006).

There are a few simulators proposed in the literature for training laparoscopic surgery’s haptic skills. These studies have focused on simulator development with haptic feedback and basic validity using standard FLS (Hamza Lup et al., 2011) part tasks. A study in the reference (Prasad et al., 2016) showed that combined visual and haptic feedback is effective in training surgeons for peg transfer and tissue dissection. The study used metrics such as peak reaction force and acceleration. Visual force feedback improved the performance of resident surgeons, and the force metrics could differentiate the experts from the residents. However, the simulation does not explain force interactions or tissue damage, nor does it simulate a complete set of FLS part tasks. The laparoscopic simulator described in (Tagawa et al., 2013) utilizes two Phantom Omni haptic devices and implements a force feedback method based on the positional error between the instructor and trainee, serving as a guide system. Evidence has demonstrated that the suggested force feedback technique enhances performance when compared to direct force feedback approaches for the cholecystectomy procedure. The study used task completion time and position inaccuracy as comparison metrics, but excluded force metrics. Furthermore, the system did not simulate and validate the procedure for a complete set of FLS tasks. The system described in (Kannangara et al., 2016) specifically focused on studying the force feedback for the palpation task. While these studies demonstrate that haptic feedback improves learner performance, the majority of systems lack force metric measurement and simulation of a complete set of part tasks for haptic skills training. Furthermore, there is a lack of explanation for simulated force interactions, tissue manipulation, or injuries in relation to the applied force. Mostly, the proficiency assessment is not based on force exertion.

The studies in (Sankaranarayanan et al., 2010; Arikatla et al., 2019) explore the face and construct validity of the virtual basic laparoscopic skill trainer (VBLaST). These studies simulated three basic tasks: peg transfer, ligating loop, and pattern cutting. They connected each laparoscopic tool in VBLaST to the haptic device (PHANToM Omni; SensAble Technologies, Inc., Woburn, MA) to provide force feedback during interactions. The average face validity score for the realism of three tasks is approximately 3.95 ± 0.909. However, for trocar placement and LapTool motions, the score is 3.67 ± 0.874. The score for the quality of haptic feedback is just 2.62 ± 0.882 on the Likert scale. Furthermore, out of the three tasks, only the peg transfer task demonstrated construct validity. A study referenced in (Sinitsky et al., 2019) examined the construct validity and virtual curriculum of the LAP Mentor II simulator (Simbionix Corp., Cleveland, OH), which has haptic feedback. They studied nine basic tasks, which included object transfer, clipping, cutting, electrocautery, camera navigation, and the appendicectomy procedure. There were no force metrics other than tissue injury (no construct; *p* = 1.000 > 0.005) to assess force skills or force feedback. The findings indicated that there was no construct validity across all parameters when comparing experts and novices. A study in (Kristine et al., 2019) explored the perception of haptic feedback in a VR simulator called LapSim^®^ VR Haptic System (Surgical Science Sweden AB, Gothenburg, Sweden) during the suturing task performed by expert surgeons. Although the haptic feedback reduces stretch damage, the realism is limited. A study (Pinzon et al., 2016) of existing VR simulators such as MIST VR, Lap Mentor II, Laparoscopy VR (Immersion Medical, Gaithersburg, MD), and LapSim showed that the haptic feedback in these simulators needs to be improved and more research is needed to make haptics realistic. Furthermore, the construct validity of these systems is inconsistent across different tasks and assessment metrics. Moreover, this underscores the need to create a customized curriculum that specifically trains haptic skills in virtual reality simulators. Therefore, there is a significant need to analyze and enhance the quality of simulated haptic feedback, as stated by (Overtoom et al., 2018). Though the studies acknowledged the necessity of enhancing the force feedback in existing systems, none of them specifically pinpointed the particular feature of haptic feedback that requires improvement or the underlying cause.

In addition to evaluating haptic skills, there is a lack of research on the realism or accuracy of force feedback, as well as the force models employed in current VR simulators. Thus, our current research aims to develop modified FLS tasks, known as haptic-based FLS (HFLS), for a customized VR laparoscopic simulator that incorporates haptic feedback. We further extend the work on improving the force model for realistic force rendering and evaluation methods.

Face validity, content validity, construct validity, predictive validity, and other validation methods are commonly used to validate the FLS part tasks and the training simulator (Schout et al., 2010; Harris et al., 2020). Face and content validity are the basic validation methods that test the simulator’s realism and whether it covers all of the relevant laparoscopic training constructs (Harris et al., 2020). Therefore, the current work validates the simulated part tasks and the simulator for basic functionality using face and content validity.

The VR training system proposed in (Kannangara et al., 2016) provides evidence supporting the benefits of using haptic feedback in a laparoscopic palpation task. The system utilizes a Phantom Omni haptic device and the Open haptics toolkit. The study uses a linear spring force model for VR object interaction. A simulator described in (Tagawa et al., 2013) has been built using a Phantom Omni haptic device and a linear force model to provide haptic feedback. The study used a mass-spring model to simulate the deformation during a gall bladder removal procedure. These studies only provided a linear model for basic deformation, not elucidating the force interactions involved in simulation. Most VR simulators with haptic feedback lack an explanation or availability of the underlying force models and force simulation methods. As a result, the accuracy, realism, and fidelity of the simulated haptic feedback have not been studied and are to be improved (Schijven and Jakimowicz, 2003; Badash et al., 2016; Pinzon et al., 2016; Aker et al., 2016). One of the reasons why the simulated force feedback is not accurate or realistic is that most of the VR simulators with haptic feedback use point-based haptic rendering (Ho et al., 1997). However, the actual LapTool has different kinetics in general compared to point-based interactions, specifically considering the laparoscopic graspers. Hence, incorporating the real LapTool’s interaction mechanism in the VR simulation may improve the force feedback. The LapTool kinetics have been studied, and the basic force model has been derived and validated through the *in vitro* experiment of a pinching task in the previous study (Susmitha Wils et al., 2017) from our research group. There is a disparity between the perceived force at the LapTool handle and the interaction force measured at the LapTool tip. The disparity is attributed to the significant scaling between the LapTool tip and the handle force, which is modeled through LapTool kinetics. In the current study, we are incorporating laparoscopic tool kinetics into the VR simulation to improve the force feedback.

Questionnaire-based evaluation in the current laparoscopic VR simulators and the force measurement in the box trainers are used to study the realism of the force feedback as discussed in (Horeman et al., 2012; Arikatla et al., 2019; Overtoom et al., 2018). The quantitative analysis of force feedback realism is lacking in the majority of simulators (Pinzon et al., 2016). Other simulators evaluate the system’s performance using metrics such as haptic rate, maximum force, z width, transparency, and the virtual environment’s maximum stiffness. These parameters quantify the simulator’s characteristics; they do not assess how well it performs for individual users. Assessing the simulator through a user-specific study will help better analyze its usability. Psychophysical techniques can be used to analyze user performance and assess the simulator.

Psychophysics allows us to measure the user’s perceptual characteristics while they engage in sensory interactions. We can use these perceptual specifications to compare simulators’ performance in various virtual reality scenarios. This is because the primary objective of any simulator is to enhance user interactions. Psychophysics models the correlation between external stimuli, such as haptic feedback, and the user’s perception of force. The just noticeable difference (JND) is a widely used perceptual metric for quantifying users’ sensory perception. The term “JND” refers to a minimally noticeable change in a user’s perception of a specific difference in the stimuli. A lower JND indicates a higher level of differential user perception. The first three chapters (Gescheider, 2013) provide an explanation of the JND and several methods for determining the perceptual thresholds for different types of stimuli. Classical psychophysics employs various models, including Weber and Fechner’s law and Stevens’ Power Law, to determine the JND (Zeng, 2020). Modern psychophysics quantifies JND as a sensitivity index using a signal detection theory and probabilistic approach (Verde et al., 2006). The VR simulations discussed in the references (Gourishetti and Manivannan, 2019; Huang et al., 2020; Prasad et al., 2016) are validated using a psychophysical experiment. To evaluate user performance in virtual simulations, these experiments measure the JND of force exertion and compare it to the JND values of real-world interactions. We can employ psychophysical approaches to examine the simulator’s performance in relation to the user.

In order to address the limitations of the VR simulators mentioned earlier, we propose HFLS-part tasks with touch feedback through our custom haptic simulator, focusing on the objective assessment of force exertion. This could help standardize the force metrics in VR haptic simulators, as FLS is already the standard. The proposed HFLS part tasks focus mainly on the application of controlled force exertion during various object manipulations, including rigid and soft bodies. We measure different force metrics and provide them as continuous, real-time feedback. We simulate the LapTool and virtual object interaction models in a way that allows the trainer to configure the proficiency force levels separately for each task. We simulate the virtual interactions based on the applied force. Therefore, we can use the proposed HFLS tasks to train laparoscopic surgery’s basic haptic skills in a VR simulator that offers haptic feedback. Further, we implement a force model of the real LapTool (grasper) in the force rendering loop of the VR simulation to enhance the realism and accuracy of force feedback, which are lacking in the available VR simulators. A LapTool tip force model is incorporated in the simulation for gripper force feedback. The effectiveness of the force model has been studied through a psychophysical experiment that quantitatively assesses the user’s perception of simulated force feedback. Whereas, the quality of simulated force feedback has been studied only via subjective questionnaires in the existing VR simulators. A laparoscopic gripping task is conducted in VR, and the force JND is measured to check whether the laparoscopic force model improves the realism of the force perception. The present investigation specifically targets the lower force range, which spans from 0.5 to 2.5 N.

The main objectives of the current work are as follows:

• We propose a set of VR-part tasks with haptic feedback (HFLS) that focus on the skills of controlled force exertion.
• Face and content validity of HFLS for a customized VR laparoscopic simulator.
• Implementing the LapTool tip force model in the VR simulation for improved gripper force feedback.
• Evaluating the realism of gripper force feedback while using the LapTool tip force model for force rendering.

In summary, the current work proposes haptic based FLS part tasks on a custom VR haptic simulator whose design highlights object manipulation through controlled force exertion and provides objective force metrics. The proposed HFLS focus on training haptic skills on a VR simulator where the applied force is included in the proficiency assessment. Further the laparoscopic tip force model is implemented to improve the accuracy and realism of the rendered force.

## 2 Materials and methods

### 2.1 Haptic part tasks

Part tasks are used to train fundamental laparoscopic surgical skills, including bi-manual LapTool interaction, depth estimation, LapTool maneuvers, hand-eye coordination, and adaptation to the fulcrum effect (Xin et al., 2006). Laparoscopic training programs around the world include a standard set of specific tasks known as FLS, offered by SAGES and the American college of surgeons (ACS) (Varras et al., 2020). In this study, we developed haptic-based FLS (HFLS) part-tasks that incorporate haptic feedback within a virtual reality (VR) simulation.

#### 2.1.1 Haptic device for laparoscopy

**FIGURE 1:**
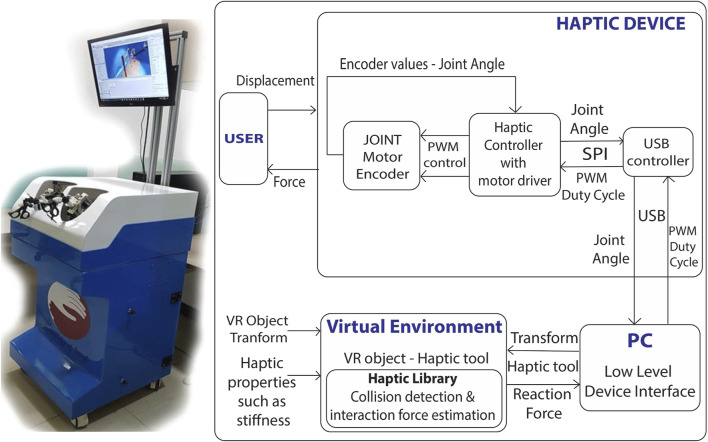
Developed laparoscopic simulator and its implementation; Left:10 DOF bi-manual VR laparoscopic simulator with haptic feedback; Each laparoscopic handle has 5 DOF motion sensing and force feedback; Right: Control architecture of haptic device; The force rendered to the user is computed continuously through the displacement measured from the haptic device.

A novel five-degree-of-freedom (DOF) bimanual haptic device has been specifically developed for laparoscopic surgical training, based on the prior design (Prasad and Manivannan, 2017). The device offers motion sensing and force feedback capabilities for various movements, including yaw, pitch, roll, in-out, and grasping motions for each hand. The haptic device functions as a serial manipulator and has modified its end effector to interface with the handles of real laparoscopic tools. This device operates based on impedance control, as described in the study (Wen et al., 2008). The device uses position as an input and force as an output. Quadrature optical encoders are used to measure each joint’s rotation angle, and actuators are used to provide torque feedback at each joint. Through the DH parameters (Denavit-Hartenberg) (Spong et al., 2020), the transformation matrix is derived for the haptic device. Through forward kinematics (Spong et al., 2020), the end effector positions are calculated for user LapTool movements in real time. Similarly, for the given interaction force from the virtual world, the torque is computed for each joint based on the Jacobian matrix (Spong et al., 2020) of the device. We then calculate the PWM (pulse width modulation) duty cycle from the torque to drive each joint actuator and produce a specific force at the end effector. An H-bridge-based motor driver (Özer et al., 2017) and the dsPIC controller boards are developed for haptic device control. We have implemented a USB communication protocol between the device controller and the PC to transfer data at a haptic rate of 2000 Hz. Figure 1 presents a functional block diagram of both the haptic device control board and the developed haptic device.

#### 2.1.2 Haptic device validation

The haptic device design is the same as in (Prasad and Manivannan, 2017) which explains the specifics of the haptic device, such as its internal design, workspace, rendering force, and other relevant information. Additionally (Prasad et al., 2016), discusses the validity of the simulator using force metrics.

#### 2.1.3 Haptic FLS

##### 2.1.3.1 Part tasks features

A total of eight laparoscopic surgical part tasks have been simulated in a VR environment. Each task focuses on the training of specific laparoscopic skills such as hand-eye coordination, LapTool manipulation, and controlled force exertion. These tasks are similar to the FLS curriculum, except each task emphasizes the application of controlled force at various ranges to complete the task successfully. Table 1 provides specific information on each of the simulated tasks. The eight tasks include a few of the extended FLS tasks, as well as a few other basic surgical tasks that are common and important across various laparoscopic surgical procedures. The peg transfer and Tower of Hanoi in HFLS are similar to the peg transfer in FLS, which focus on the LapTool maneuvers through rigid body interactions. The difference in the current implementation is that it monitors and records the interaction force during collisions, grabbing, and dropping of objects. The force applied during the displacement of the object beyond the field of vision is also quantified. We then compare the values with the reference force values defined by the trainer. The objects in the peg transfer have joints at the corners that break if the user applies too much pulling force. The properties of the peg and objects can be modified to simulate various interaction scenarios with diverse force feedback. Similarly, cloth cutting and rope transfer tasks are nearly identical to FLS tasks, which focus on cutting and maneuvering elastic objects. The difference lies in monitoring interaction forces while picking, pulling, and pushing. Excessive pulling will cause VR objects such as rope and cloth to tear apart. Additionally, clipping and electrocauterization are considered since they are very common steps performed in most laparoscopic surgeries. Other than these tasks, two more are considered to highlight the application of controlled force: ring transfer and handling of irregular soft body objects. Both tasks involve applying controlled directional forces while twisting and translating objects. In addition to force, all tasks also emphasize hand-eye coordination, depth perception with haptic feedback, the fulcrum effect, and LapTool dexterity. Given that box trainers in FLS training provide real-force feedback from objects, it is imperative to incorporate haptic feedback into virtual FLS tasks. The implemented HFLS part tasks enable the trainer to assess the forces involved in interactions in real-time. The trainer can modify the interaction parameters to alter the level of difficulty of the tasks.

**TABLE 1:** HFLS Part Tasks: Skills and Assessment Metrics; Metrics related to haptics are highlighted in bold, where for each metric, the corresponding applied force is measured and compared with the trainer’s specified force threshold to increment the error.

Task	Description	Assessment parameters
Common to all task		1. Time
		2. **Mean force**
		3. **Peak force**
		4. Maximum velocity
Peg transfer	This task focuses on the bimanual handling of objects with less force. The trainee has to transfer the object from a non-dominant hand to another hand in mid-air and place it in the specified location. The same has to be repeated for the dominant hand. The objects are simulated as very lightweight rigid body objects, so applying too much force will make the objects move with high velocity. The trainee has to apply a controlled force to complete the task	1. **Number of drops**
		2. Number of complete and incomplete transfers
		3. **Number of objects dropped out of view**
		4. **Number of collisions with the path objects**
Tower of Hanoi puzzle	This task focuses on the bimanual handling of objects with curvilinear edges. The trainee has to transfer the objects from the first rod to the last rod in the same order. The game has to be finished with a minimum number of moves and less time. The trainee has to apply a controlled force to grab the rings or else the rings will slip from the grasper	1. **Number of drops**
		2. Number of transfers
		3. **Number of objects dropped out of view**
		4. **Number of collisions with the path objects**
Ring transfer	This task focuses on using the LapTool’s twisting, pulling, pushing, and gripping functions simultaneously. A consistent amount of force and movement has to be applied in all directions to grasp the object firmly. The trainee has to traverse the ring through the curved path without colliding with the loop by each hand. Removing both rings will complete the task	1. **Number of drops**
		2. **Number of collisions with the path objects**
Clipping the soft body	This task focuses on the ability to clip the soft body object precisely at the marked positions. Clipping at both marks simultaneously with each handle will complete the task	1. Distance error in clipping
		2. **Clipping force**
Rope transferring	This task focuses on the ability to handle an elastic object in a curved and complex path. The trainee has to traverse the rope from one end to the other end of the loop without tearing it	1. **Number of rope cuts**
Electro-cauterization of strings	This task focuses on using the bimanual Lap handle with the electro-cauterization tip to precisely cut the elastic strings at the marked positions	1. **Number of cuts at wrong positions and strings**
Cloth pattern cutting	This task focuses on cutting the elastic cloth at the predefined circular path using both Lap handles. The task requires the use of very controlled and precise movements	1. **Number of wrong cuts**
Handling irregular objects	This task focuses on the bi-manual handling of the irregularly shaped soft-body object. The trainee has to orient the soft body to the target position without dropping it. The task requires using significantly less amount of controlled force to avoid the jumping and slipping of the object	1. **Number of drops**

##### 2.1.3.2 Simulation tools

**FIGURE 2:**
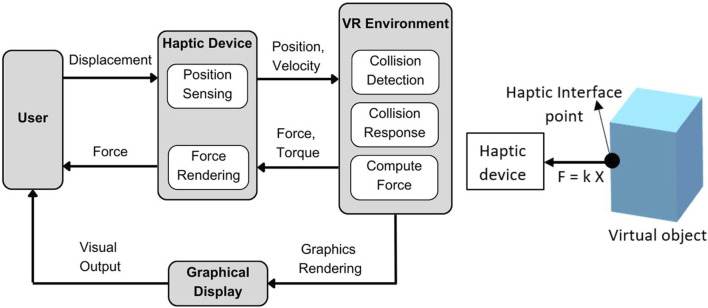
Overview of force rendering in the simulator; Left: Overview of user VR interaction; where the user position is tracked through a haptic device and updated in VR for collision detection. The reaction force is calculated and rendered to the user via a haptic device; Right: Force calculation for LapTool-object interaction. The force is calculated based on the collision between the haptic interface point (HIP) and the virtual object.

The tasks are simulated using the Unity 3D game engine and physics libraries such as Nvidia Flex and OBI, which are used to simulate soft bodies using the PBD (position-based dynamics) algorithm (Müller et al., 2007). The Unity3D graphics rendering system is utilized for the visual rendering of the simulation. When the user interacts with VR objects, the haptic device renders a continuous interaction force. The user can perceive the shape, size, curvature, weight, texture, and collision of the VR objects through the force feedback. Figure 2 outlines the basic flow of user interaction with the VR object. This gives an overview of the functions within the haptic rendering loop. The haptic device senses the user’s position as they manipulate the end-effector, mapping it to the virtual LapTool. The haptic device calculates and renders a reaction force to the user through the end-effector based on the collision detection between the virtual LapTool and the object.

##### 2.1.3.3 Virtual laparoscopic tool - Object interactions

In VR, each HFLS task simulates various interactions between objects and the LapTool. One of the main virtual LapTool-object interactions is the gripping and clipping of the VR object. The gripping and clipping are simulated through a position constraint on the object. We extend the haptic collision detection and finger proxy algorithms (Talvas et al., 2013) in VR to support two LapTools. The force rendering of the basic virtual LapTool interactions with rigid bodies is achieved using a simple linear force model (F = kX), as shown in Eq. 1. The force calculation is derived from the God-object technique (Talvas et al., 2013) and is presented in Figure 2. We employed a basic piecewise linear model to represent complex interactions, such as gripping, cutting, and clipping.

$$F\left(x\right)=\left\{\begin{array}{cc} 0\; & x<x_{c}\\ kX\; & x>x_{c} \end{array}\right.$$ (1)

where *x* is a LapTool pose, *x*_*c*_ is the LapTool contact position with the VR object, *X* is the distance between the goal and proxy position of the haptic interface point (HIP) (Talvas et al., 2013; Conti et al., 2005), and k is the user-defined object stiffness given by the equivalent stiffness of a material. The HIP is a virtual interaction point that maps the actual haptic device transformation in the virtual environment, as shown in Figure 2. Eq. 2 provides the force function for the gripping, clipping, and cutting of the VR object.

$$F\left(x\right)=\left\{\begin{array}{cc} 0\; & x<x_{c}\\ kX+\left(kX+b\dot{X}\right)u\left(x-x_{c}\right)\; & x>x_{c},F<F_{T}\\ F\; & x>x_{c},F>F_{T} \end{array}\right.$$ (2)

where *F*_*T*_ is the threshold force defined by the expert surgeon, *b* is the damping coefficient, and $\dot{X}$ is the velocity of the LapTool. The {*u*(*x*) : *xϵR*} is the step function. *F* = *F*_*T*_, for gripping, whereas *F* = 0 for clipping and cutting. The clip gets attached to the object, and the cloth material gets cut when the force is greater than the threshold force. The threshold force is different for gripping, clipping, cutting, and electro-cauterization.

**FIGURE 3:**
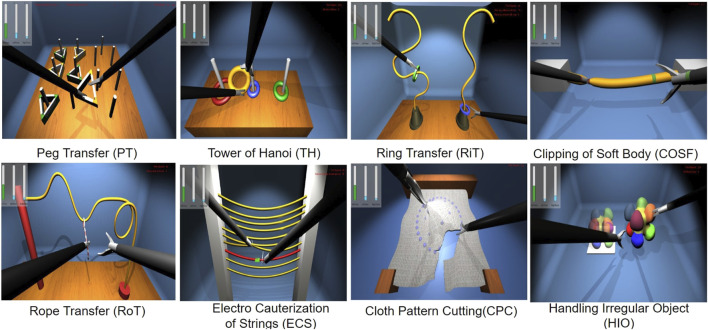
Laparoscopic haptic part tasks with touch feedback; where the laparoscopic tool is used to interact with virtual objects of each task for transferring, clipping, traversing, electro-cauterizing, cutting, and gripping.

The force function for electro-cauterization is described in Eq. 3.

$$F\left(x\right)=\left\{\begin{array}{cc} 0\; & x<x_{c}\\ kX\; & x=x_{c}\\ F_{T}\delta \left(x-x_{c}\right)\; & x>x_{c} \end{array}\right.$$ (3)

where {*δ*(*x*) : *xϵR*} is the impulse function. The HFLS part tasks simulated are shown in Figure 3.

##### 2.1.3.4 Assessment

During task performance, we measured assessment parameters such as time, force, and error in real time for each task. We displayed these parameters as real-time feedback to the trainee. At the end of the performance, we also display a scoring report based on the assessed parameters. Table 1 provides the details of each task, the skills learned, and the assessment metrics measured for each task. The part-tasks are designed in such a way that the trainee can complete the task only if they apply a controlled amount of force and velocity while interacting with VR objects. The trainer can alter the threshold for interaction force and velocity for each task. For instance, the trainer can adjust the force threshold for the LapTool’s gripping function, which simulates the gripping of an object based on specified object properties like stiffness and the threshold force value. The reaction force of each LapTool is measured and displayed in real-time to the trainee.

#### 2.1.4 Haptic VR interface

**FIGURE 4:**
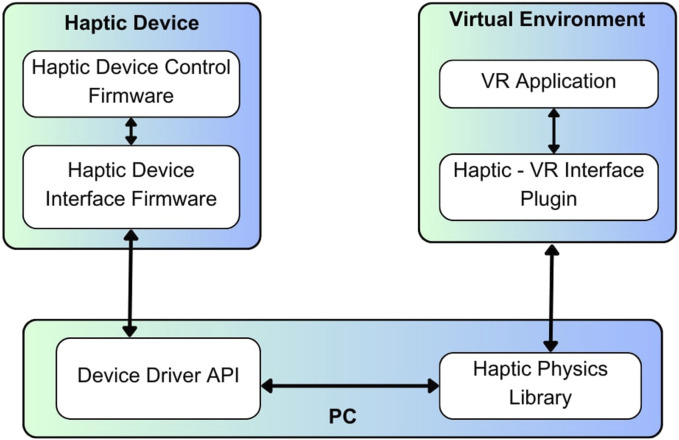
Architecture of software implementation; where the software layers are shown for haptic device control, VR environment interface, and the haptic rendering.

A customized 10-DOF haptic device is interfaced with the HFLS-part tasks for interaction. A CHAI3D haptic library is utilized for haptic collision detection and force rendering, as discussed in (Conti et al., 2005). We have extended the haptic library to accommodate custom LapTool interactions that are necessary for laparoscopic tasks. A custom VR haptic plugin has been developed to interface the haptic device and the haptic library with the HFLS task simulation. We primarily use the plugin to interface VR with the haptic rendering loop and the haptic device. This plugin tracks the position of the device’s end effector and applies force to the haptic device. It also estimates the HIP force and movement assessment parameters for each laparoscopic tool. Figure 4 shows the software architecture of the VR-haptic interface. The haptic device comprises two software layers. One layer is responsible for controlling the actuator to provide force feedback, while the other layer facilitates a full-duplex connection with the PC for data transfer. On the PC side, there are two software layers that operate at a low level. One layer is responsible for communicating with the haptic device, while the other layer runs a haptic rendering loop that connects the device to the VR application. The VR application consists of two software layers. The first layer interfaces with low-level haptic libraries, while the second layer runs the high-level VR program and allows for customized simulation rendering. Each of the several layers operates simultaneously using RTOS (real time operating system) functionalities, multi-threads, and kernel resources to synchronize and achieve a faster haptic update rate. We utilize RTOS to implement event-driven and time-shared multitasking processes with priority scheduling (Zorkany et al., 2016).

### 2.2 Face and content validity

The developed laparoscopic part tasks simulation is evaluated using face and content validity (Schout et al., 2010; Harris et al., 2020). Face validation tests assess the appropriateness of the test material for its intended purpose. Simply put, face validation determines the simulator’s degree of realism in relation to a real-life surgical procedure. The process of determining if a test adequately covers all the different features of the measure it intends to assess is known as content validation. Put simply, it assesses if the simulator possesses all the essential functionalities required for laparoscopic surgical training. Most of the existing laparoscopic simulators, such as Laps, MIST VR, Lap Mentor II, and VBLaST given in (Pinzon et al., 2016), were analyzed through face and content validity as initial verification before construct validity. Even though the face and content validity are subjective measures collected through a set of questionnaires (Leijte et al., 2019), it is one of the most commonly used and basic tests to start with (Schout et al., 2009; Leijte et al., 2019; Esteban et al., 2022). Based on the literature study, it was observed that there are no standard questionnaires for face and content validity, and they vary among different simulators, curricula, and studies. Hence, in the current study, we tried to cover major parts of the existing questions regarding the functionalities of the LapTool, highlighting the haptic aspects. Face validity is kept simple to compare the realism of each simulation component with the actual surgery.

We used a five-point Likert scale as explained in (Gumbs et al., 2007) as a measurement tool, where participants are requested to rate their responses on the questionnaire. The rating scale varies from “strongly disagree” to “strongly agree,” with values ranging from one to five and an inter-option spacing of one. The participant has to perform eight simulated tasks and respond to a questionnaire that assesses the simulator’s reality and functionality for each task. The sample questionnaire used in the validation experiment is given in Table 2. We first asked the novice subjects to perform a few tasks, including transferring rings and gripping soft objects in a simple box trainer, to become familiar with the LapTool. We used this as a baseline for the novice to evaluate the realism of the VR simulation.

**TABLE 2:** Sample face and content validity questionnaire.

Question - face validity	Question - content validity	Likert score (1–5)
Do the handle tools shown in the test look like an original Lap handle used for picking an object?	Did the dexterity of handling Laparoscopic tools feel realistic?	
Does the peg board shown in the test look like an original peg board?	Did the picking of objects with a Lap handle feel realistic?	
Do the pegs shown in the test look like the original peg rod?	Did the haptic feedback for the objects feel realistic?	
Does the triangular block objects shown in the test look like the original triangular blocks?	Did the presence of haptic feedback improve the learning experience?	
	Does the device workspace feel realistic?	
	Did the scaling factor between the physical to virtual environment feel realistic/comfortable?	
	Were you able to do the task without any assistance?	
	Were the parameters helped to improve your learning curve?	

#### 2.2.1 Subjects

Thirty-two healthy subjects performed eight simulated tasks and answered the face and content validity questionnaire, which covers the realism and functionality of the developed laparoscopic simulator. They reported no perceptual or psychomotor learning disorders. The subjects are aged 21–53 years (mean of 30 years and standard deviation of 9 years) with an average weight and height of 67 ± 11kg and 168 ± 7 cm, respectively. The experiment included participants of both genders, with twelve of them being female. Each participant provided informed consent. Despite being right-handed, all the subjects performed the laparoscopic part tasks with both hands. Among the subjects, five are the most experienced, five have moderate experience, and the others are beginners. Subjects with more than 5 years of experience are considered experts, while those with 2 years of experience are considered intermediate, and those with no hands-on experience are considered novices. The protocol of the studies involving humans is approved by the Institutional Ethical Committee (IEC) of IIT Madras (IEC/2023-03/MM/01/02). The study conforms to the standards set by the latest revision of the Declaration and follows local legislation and institutional requirements. Informed consent is obtained from all participants involved in the study for the publication of the data.

### 2.3 Laparoscopic tool force model

Expert laparoscopic surgeons provide significant feedback through face and content validity, indicating that while haptic feedback improves learning performance, its realism can be enhanced. The characteristics and nature of the interaction of the real laparoscopic tool (LapTool) differ from the point-based interaction implemented in the part-task simulation (Ho et al., 1997). Hence, to improve the realism of force rendering, the LapTool kinetics are considered and implemented in the simulation. We integrate the LapTool’s force model into the force rendering loop, adding it to the current force model to determine the gripping force. We conducted a psychophysical experiment to evaluate the impact of incorporating the new LapTool force model into the force rendering on the user’s haptic perception.

#### 2.3.1 Force model implementation

##### 2.3.1.1 Gripper force estimation

**FIGURE 5:**
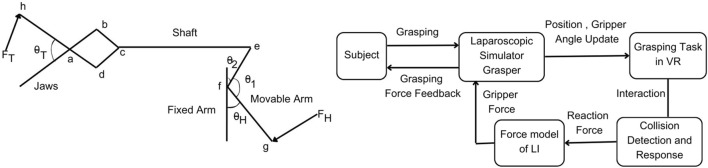
Overview of force model implementation; Left: Kinetics of double jaw action laparoscopic grasper adapted from Susmitha Wils et al. (2017), where *F*_*H*_ is the handle force, *F*_*T*_ is the tip force, *θ*_*H*_ is the handle angle and *θ*_*T*_ is the tip angle; Right: Overview of the LapTool force model implementation; where the LapTool force model is applied over the calculated reaction force to derive the gripper force at the laparoscopic handle.

In the literature, the force model of the real LapTool tip was referred to as (Susmitha Wils et al., 2017) and incorporated into the force feedback rendering pipeline of the simulation. This study only focused on the gripping task and force model of the double jaw action laparoscopic grasper, as it is the same grasper used in the simulator. The kinetics of the LapTool given in (Susmitha Wils et al., 2017) are shown in Figure 5. Through deriving the moment balance equation at various joint positions along the LapTool, the grasper’s tip force is determined as given in Eq. 4.

$$F_{T}=\left\{\begin{array}{cc} \frac{\left(F_{H}-F_{L}\right)\times fg\times ad}{2fe\times ah\;cos\left(180-\left(\theta_{H}+\theta_{1}\right)\right)cos\left(\frac{\theta_{T}}{2}\right)},\; & \alpha =90{}^{\circ}\\ \frac{\left(F_{H}-F_{L}\right)\times fg\times ad\;sin\alpha }{2fe\times ah\;cos\left(180-\left(\theta_{H}+\theta_{1}\right)\right)cos\left(\frac{\theta_{T}}{2}\right)},\; & \alpha <90{}^{\circ}\\ \frac{\left(F_{H}-F_{L}\right)\times fg\times ad\;cos\left(\alpha -90\right)}{2fe\times ah\;cos\left(180-\left(\theta_{H}+\theta_{1}\right)\right)cos\left(\frac{\theta_{T}}{2}\right)},\; & \alpha >90{}^{\circ} \end{array}\right.$$ (4)

where *F*_*T*_ and *F*_*H*_ refer to the forces at the LapTool’s tip and handle, respectively. The *F*_*L*_ denotes any loss in force transmission between the grasper’s handle and tip. If *F*_*H*_ is zero, then *F*_*L*_ becomes zero, since the loss due to force transmission at the joints is zero. The parameters *fg*, *ad*, *fe*, and *ah* are given in Figure 5, referring to the different moment arms at joints. The angles *θ*_*H*_ and *θ*_*T*_ correspond to the angles at the handle and tip, respectively. *θ*_1_ refers to the angle at joint *f* shown in Figure 5, and angle *α* is given by *α* = 180 − *θ*_*T*_. In order to incorporate the LapTool tip force model (Susmitha Wils et al., 2017) into the simulation, we consider the reaction force resulting from the virtual HIP as the LapTool tip force. The force at the handle is then determined using the LapTool tip force model, as described in Eq. 4. We modified the API for the custom haptic device driver to calculate the gripper torque by multiplying the handle force with the moment arm length. The moment arm length is the distance from the gripper tip to the joint location denoted as *fg* in Figure 5. The gripper of the haptic device is actuated to apply the calculated torque. Eq. 5 specifies the gripper joint torque.

$$T_{g}=fg\times F_{H}$$ (5)

where the term *T*_*g*_ represents the torque applied to the gripper joint. The experimental regression model from (Susmitha Wils et al., 2017) is used to figure out the gripper tip angle and force loss in this implementation, as shown in Eqs 6, 7.

$$\theta_{T}=6.835\;\theta_{H}$$ (6)

$$F_{L}=0.111\;\theta_{H}-0.776$$ (7)

##### 2.3.1.2 Force model - VR integration

**FIGURE 6:**
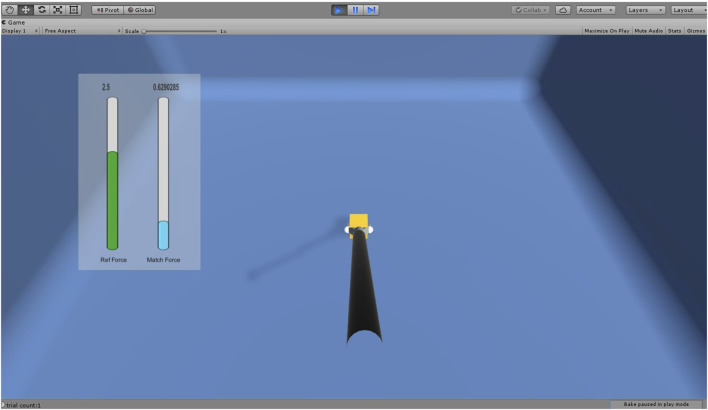
Laparoscopic gripping task for force JND experiment where the force is applied over the cube using LapTool gripper. The reference force is shown in green color in the slide bar. The applied force is shown in the slider bar in blue color if it is less than the reference force and in red color when it is greater than the reference force.

The overview of the force model implementation is given in Figure 5. The gripper angle is tracked continuously through a sensor attached to the handle and used to update the LapTool grasper angle in the VE. Based on the collision with the virtual object, the force at the gripper HIP point is calculated from the finger proxy algorithm as discussed in Section 2.1.3. From the calculated gripper reaction force, which is the tip force, the actual handle force is derived from the tip force model given in Eq. 4. The gripper torque derived from the handle force using Eq. 5 is then utilized to render the gripper force feedback to the user. We designed the gripping task using the Unity 3D game engine. The custom haptic-VR interface plugin was modified to extend a two-point haptic interaction from CHAI3D (Conti et al., 2005) for the gripping task. CHAI3D haptic libraries and Bullet physics (Coumans, 2015) libraries were extended and used to implement the force feedback for the gripping mechanism. We interfaced the designed task with our custom 10-DOF laparoscopic simulator for force feedback. The designed gripping task is given in Figure 6, which shows the simple gripping of a static cube in VR.

In the virtual environment (VE), the haptic device typically maps to a specific point (HIP), and a linear force model calculates the force of interaction between the point and the virtual object. The reaction force is directly proportional to the displacement of the point and is scaled by the object’s stiffness. This force is utilized to compute the gripper torque and provide force feedback. However, in the actual laparoscopic tool, the interaction occurs between the grasper and the object via a 4-bar mechanism and the shaft connecting the tip and handle. The tip force model captures the potential force scaling that occurs between the grasper tip and handle. Incorporating the tip force model into the virtual simulation resolves the issue of scaling and accounts for any force reduction caused by friction. The force model enhances the accuracy of force rendering, making it comparable with the real laparoscopic grasper. This has the potential to impact the force that the surgeon senses and applies during object interaction in training, thereby affecting their haptic skills.

### 2.4 Force JND experiment

In order to study the effectiveness of the gripper force feedback with the LapTool tip force model, we conducted a psychophysical experiment to assess the user’s perception of force. The study aims to determine if there is a reduction in the force JND when utilizing the LapTool tip force model for gripper force feedback. The JND is employed to quantify the perceptual sensitivity of the sensory system. A lower JND indicates greater system sensitivity, leading to improved user perception and performance. The study employed the psychophysical method known as the constant stimuli method (Gescheider, 2013). This method involves presenting stimuli randomly and independently. We employ the psychophysical method to investigate the verisimilitude of force feedback by examining the user’s perception of a specific psychophysical measure, such as the JND of force. We are evaluating the accuracy of force rendering caused by the LapTool tip force model by examining user force perception.

#### 2.4.1 Experiment design and protocol

For the study, the force matching task was considered (Gourishetti and Manivannan, 2019). We instructed the subject to place the LapTool and exert a grip force along the sides of the static and rigid VR cube to match the specified reference force. We recorded the matching force values for 2 minutes at a sampling frequency of 60 Hz. Five reference force levels considered for the task are 0.5N, 1N, 1.5N, 2N, and 2.5N, with a constant interval of 0.5N. The allowable range of force variation was 0.025 N. We provided a 1-min break after every 20 trials and a 5-min break between two distinct force feedback conditions to mitigate the influence of muscle memory and other subjective biases. The experiment consists of two parts. The first part of the experiment exclusively utilizes linear force models for force feedback, while the next part incorporates the tip force model. We randomly varied the sequence of the two experiment conditions across participants to prevent any potential bias resulting from the order. The experimental setup remains consistent across both conditions, except for gripper force feedback. The experiment involved twelve healthy individuals who had no prior experience or training in laparoscopic surgery. For the simplicity of the analysis and since the subjects were novices, only the dominant (right) hand was considered for the experiment. The GUI progress bars provided visual feedback for the applied forces. A total of 100 trials were conducted, with ten trials for each force level across two separate experimental conditions. The experimental scene is composed of a stationary VR cube and a laparoscopic grasper, as shown in Figure 6. The vertical bars display the applied and reference force values individually. The reference force is shown in green. When the applied force exceeds the reference force, the display turns red, and when it falls below it, it turns blue. A green color denotes the exactness of both the applied and reference forces.

#### 2.4.2 Subjects

Twelve healthy subjects performed the gripping tasks for two conditions of the experiment: one with force feedback using the LapTool force model, and one without the force model. They reported the absence of any perceptual or psychomotor learning disorders. The participants in the study range in age from 25 to 48 years, with a mean age of 29 years and a standard deviation of 6 years. On average, their weight is 68 ± 10 kg, and their height is 172 ± 8 cm. The experiment featured volunteers of both genders, with five of them being female.

#### 2.4.3 Measures

We computed psychophysical measures from the recorded force values, such as the percentage of absolute force JND (Gescheider, 2013) and the coefficient of variation (COV) of force. The JND is the smallest change in a stimulus that is required to produce a noticeable difference in human perception. This study focuses on the JND of exerted force control rather than the perception of force. This is a metric that quantifies the accuracy of perceiving and controlling forces. Eq. 8 is used to calculate the absolute JND based on the exerted and reference forces.

$$\left(\%\right)AbsoluteForceJND=\left(|F_{m}-F_{r}|/F_{r}\right)*100$$ (8)

where *F*_*m*_ and *F*_*r*_ (>0) are referred to as the applied matching force and the given reference force values.

COV is a measure of force variability, defines the accuracy of the force value, and is calculated as in Eq. 9.

$$\left(\%\right)COV=\left(F_{\sigma }/F_{\mu }\right)*100$$ (9)

where *F*_*μ*_ and *F*_*σ*_ are the mean and standard deviation of the applied matching force.

We calculated the mean and standard deviation of the absolute force JND and COV by averaging the force values across all trials and participants for each force level. A one-way ANOVA was conducted to determine the significance of each force level and the overall mean force value for two distinct force feedback methods in the experiment.

## 3 Results

### 3.1 Face and content validation

**FIGURE 7:**
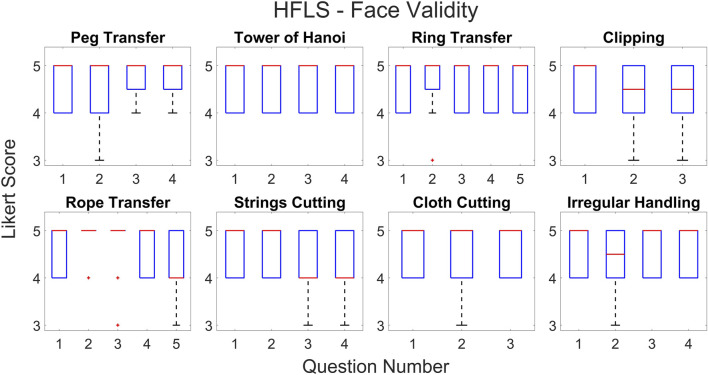
Box plot showing Likert score for each question and task for face validation.

According to the statistical results, the majority of questions in all HFLS tasks have a Likert score of four or higher. We have analyzed the experimental data for face and content validation using statistical metrics like mean, median, standard deviation, variance, and quartiles. Table 3 presents the comprehensive scores for both the face and content validity of each task. The results indicate that the average Likert score for all HFLS tasks in face validity is higher than 4.6. Similarly, in terms of content validity, the average Likert score exceeds four for all of the part tasks. A score of four on the Likert scale indicates agreement. The face validity exhibits a mean variance below 0.3, whereas the content validity demonstrates a mean variance below 0.5. Figure 7 depicts the box plot representing the Likert score for each task and question in face validation. All questions have a median and 75th percentile score of 4.5 or higher, as well as a 25th percentile score of four or above. On the Likert scale, a rating of four or five corresponds to the responses “agree” and “strongly agree,” respectively. All HFLS tasks received the lowest minimum score of three or above, corresponding to the response option “Neither Disagree nor Agree.” Tasks such as clipping, rope transfer, string and cloth cutting, and irregular handling got a minimum score of 3. The question “Does the gray board shown in the test resemble an original metal or wood support board?” of the clipping and cutting task has the lowest minimum score. This question pertains specifically to the supporting component and does not influence the overall realism of the manipulated object. In other tasks, the focus of the questions was on the appearance of soft body objects, including ropes, strings, and squeezable objects, along with the sparks produced during electro-cauterization. Furthermore, the rope transfer task has a distinct outlier score of three in relation to the question about the realism of the supporting metal rods. Once again, the question relates to the stationary objects in the background rather than the manipulating objects. In this case, the outlier is negligible, as the median score is 5. The simulation’s overall median score of 4.5 or higher shows that it was generally considered realistic. However, the minimum score highlights specific areas for improvement, such as the modeling of soft bodies.

**TABLE 3:** Face and Content validation for each HFLS part tasks with a Likert scale of five.

Tasks	Average mean (out of 5 on likert scale)		Average variance	
	Face validity	Content validity	Face validity	Content validity
Peg Transfer	4.846	4.422	0.179	0.384
Hanoi Tower	4.827	4.418	0.154	0.425
Ring Transfer	4.769	4.385	0.223	0.476
Clipping soft body	4.692	4.351	0.274	0.446
Rope Transfer	4.862	4.382	0.192	0.471
Strings cauterization	4.788	4.441	0.218	0.420
Cloth Cutting	4.744	4.353	0.248	0.494
Handling Irregular object	4.885	4.438	0.103	0.415

**FIGURE 8:**
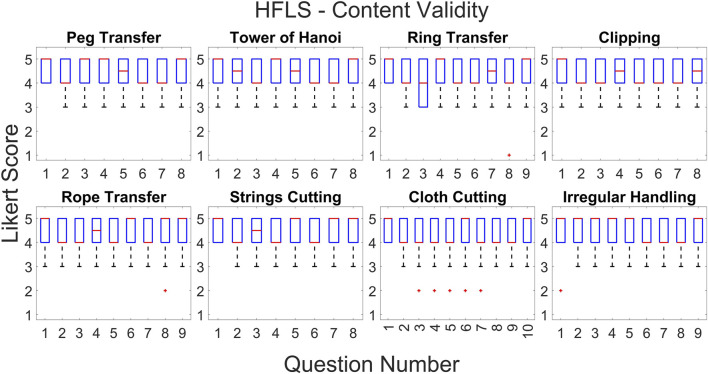
Box plot showing Likert score for each question and task for content validation.

The quartiles range from four to 5, with a minimum score of three for all tasks and questions pertaining to content validity. Figure 8 depicts a box plot illustrating the Likert score for content validation. The plot displays the scores assigned to each task and question, which characterize the simulator’s function. The median and 75th percentile scores for all task questions range from 4 (agree) to 5 (strongly agree). The 25th percentile score for all questions is 4, except for the third question in the ring transfer task, which received a score of 3 (“Neither disagree nor agree” on the Likert scale). The third question in the ring transfer task is, “Is it feasible to manipulate the ring along the path while considering lap handle movement?”. The low score for the ring transfer task may be attributed to the higher level of difficulty associated with the task. In order to effectively complete the task, the user must possess the ability to maneuver the ring in all degrees of freedom of motion without any collisions with the center rod or accidental detachment. Only experienced surgeons are more likely to succeed in executing this task during their initial attempts. Consequently, novice surgeons might assign a lower rating to this particular task, especially if they receive it during the initial trials. However, further analyses of expert scores can confirm this. The majority of task questions have a minimum score of 3, indicating that a large number of participants have accepted the simulator functions. There is a single outlier with a value of two for questions related to cloth cutting, rope transfer, and irregular soft body handling. These outliers are for novices who have difficulty with the task, particularly when it involves complex manipulation using both hands. The simulator’s functionality has received an overall median score of over four for all tasks and questions, indicating general approval of it. The minimum score of three could be attributed to the perceived task difficulties of novices or the limitations of the simulator. Examining the results based on the subjects’ level of surgical expertise will provide a further explanation.

**FIGURE 9:**
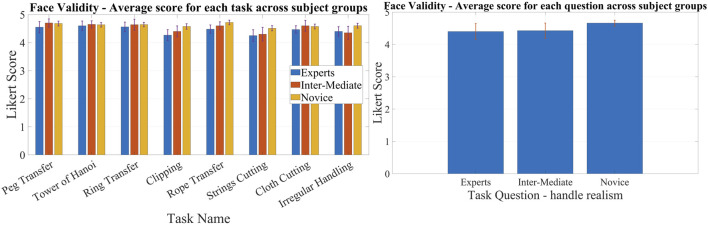
Comparison of face validation results among subject groups of different laparoscopic experience; Left: Comparison of average Likert score between different subject groups for each task for face validation.; Right: Comparison of average Likert score between different subject groups for a common question for face validation.

Face validation results for complex surgical tasks vary among novice, intermediate, and expert groups, while scores for other tasks are comparable. The face validation findings are displayed in Figure 9, illustrating the scores given by various subject groups for distinct tasks and a common question. The scores in Figure 9 are obtained by averaging the results across questions and subjects within a certain group and are presented for each task. Each subject group gave nearly identical scores on basic manipulation tasks, including peg transfer, tower of Hanoi, and ring transfer. Nevertheless, there was a noticeable disparity in scores among the subject groups when it came to intricate tasks like clipping, rope transfer, and string cutting using electrocauterization. These tasks require precise softbody manipulation and specialized surgical skills. Experts scored lower than intermediates, who in turn scored lower compared to novices in all of the tasks. However, both novices and intermediates gave almost identical scores, and they were higher than the experts in cloth pattern cutting. When it came to irregular handling, experts and intermediates scored similar and lower than the novices. The experts gave scores lower than the novices for all the tasks. This could be because the experts have more real-life experience compared to the novices. However, the novices underwent initial trials using box trainers and were referred to real surgical videos for comparison. In general, all groups gave a score of 4 (“agree”) or higher, indicating that the subjects, regardless of their surgical experience, perceived the simulated tasks as realistic. Figure 9 displays the mean scores of various subject groups for a common question: “Do the handle tools shown in the test look like an original Lap handle used for object picking?” across different tasks. All groups gave a score higher than 4, with intermediates and experts giving scores slightly lower compared to novices.

**FIGURE 10:**
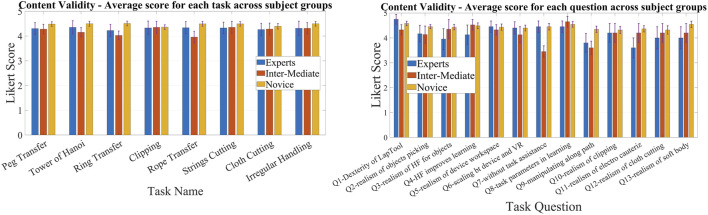
Comparison of content validation results among subject groups of different laparoscopic experience; Left: Comparison of average Likert score between different subject groups for each task for content validation.; Right: Comparison of average Likert score between different subject groups for each question for content validation.

Experts and intermediates rated the tasks lower in content validation compared to the novice group. The results of content validation for each subject group are shown in Figure 10. Figure 10 presents the scores averaged over questions for different tasks, categorized by specific subject groups. The scores for all the tasks were lower among the experts and intermediates compared to the novices. This may be due to the fact that experts and intermediates have more practical knowledge than novices and can critically assess it. However, novices can only compare tasks using the box trainer as their reference system. On the other hand, experts and intermediates utilize actual surgical procedures as a benchmark for comparison. Thus, novices may give higher ratings compared to experts. To put it simply, the simulator’s performance is comparable to that of the box trainer, but it needs improvement when compared to real surgical manipulation. Although the expert scores surpass 4, signifying their acceptance of the simulator’s functionalities, we can examine the slight difference between experts and novices to improve specific tasks. The ring transfer and cloth cutting tasks received a score of 4.2, the lowest among the tasks, according to the experts. For the other tasks, the score was approximately 4.3. Both of these tasks require the use of both hands to maneuver objects, particularly with complex twisting movements along a curved path. The disparity in results between experts and novices is more pronounced in the tasks of peg transfer (4.08%), ring transfer (6.28%), and irregular soft body handling (4.09%). The discrepancy for the remaining tasks is approximately 3%, except for clipping, which has a discrepancy of 0.76%. Similar to ring transfer, irregular handling involves twisting the soft body object to align with the target location. When it comes to peg transfer, it is crucial to align the graspers so that they can securely hold the triangle object without any slippage. These observations indicate that the simulator’s twisting function requires improvement. The scores of experts and intermediates were generally comparable across most tasks, with the exception of a few, namely, the Tower of Hanoi, ring transfer, and rope transfer. Intermediates gave a lower score than experts on these tasks. Each of the three tasks requires advanced object manipulation, simultaneous multidirectional movement, and precise force control to prevent objects from dislodging from their intended path. The possibility exists that the intermediates found the tasks more challenging compared to the experts, resulting in lower scores on those particular tasks. One possible factor contributing to this distinction is the level of force applied when handling the objects, particularly the force required to prevent slippage. According to (Prasad et al., 2016), intermediates exert a greater amount of force compared to experts. All subject groups had nearly identical performances on the clipping task. This could be because the task is not very challenging. The clipping task focuses primarily on gripper control and does not require much manipulation. Additionally, the subjects can perform the task sequentially, concentrating on one hand at a time. As a result, the clipping task is a straightforward task that only evaluates the simulator’s gripper functionality. The findings suggest that the gripper of the simulator exhibits comparable performance to both the laparoscopic tool employed in real surgery and the gripper of the box trainer. Subjects from all three groups rated all simulated tasks with a score of four or higher, indicating that subjects with varying levels of surgical experience found the simulator’s functionality satisfactory.

The content validation of the questions across groups and tasks reveals that experts assign lower ratings to certain questions compared to novices. However, for the other questions, the results are similar. Figure 10 displays a comparison of the Likert scores provided by different subject groups for content validation. We compared the scores for each common question by averaging across the tasks. Such a comparison would offer a more precise perspective on how the subjects perceive the functionalities of the simulator. The experts gave lower scores than the novices for most of the functionalities, such as the realism of picking (Q2), haptic feedback (Q3), its effect on learning (Q4), manipulation (Q9), clipping (Q10), electrocauterization (Q11), cloth cutting (Q12), and soft body interaction (Q13). In contrast, both experts and novices gave similar scores to questions about the realism of the device workspace (Q5), scaling between the device and VR environment (Q6), task assistance (Q7), and task parameters (Q8). It is reasonable for experts to give lower scores because they have more practical knowledge and can compare the simulator’s functions to those of actual surgery. On the other hand, novices can only compare the simulator’s functions to those of a box trainer, and they may give higher scores than experts. It is interesting to note that the difference in score between experts and novices is only evident in complex surgical tasks such as grasping, clipping, cutting, manipulation, haptic feedback sensing, electrocautery, and soft body interactions. However, when it comes to general LapTool functions, such as workspace and VR scaling, the score is almost identical. The observation corroborates the hypothesis pertaining to the reference system used for the purpose of comparison. According to the results, the basic functions of the simulator LapTools are comparable to those of actual surgical LapTools and box trainers. However, for more complex LapTool functions, the simulator is similar to the box trainer and requires improvement when compared to actual surgery. It is worth noting that experts gave a higher score than novices for LapTool dexterity, indicating that the simulator LapTool may be superior to the box trainer. We can use the disparity between the scores of experts and novices to pinpoint the simulator functions that require enhancement. The expert assigned the lowest scores to the following functions: realism of haptic feedback (3.95), manipulation (3.8), and electro-cautrization (3.6). As previously discussed, there is a need for improvements to the twisting function and electro-cauterization sparks. After analyzing the outcomes of each task, we can conclude that improving the haptic feedback for cutting and gripping, particularly during manipulation, is necessary. The expert assigned a score of 4, signifying the need for improvements in the realism of cloth cutting and soft body interactions. However, all other functions received a score higher than 4. The realism of the simulation, particularly the aspects discussed above, is primarily responsible for the reduced expert score.

The scores for the majority of LapTool functions in the intermediate group were lower compared to the novice group, with the exception of Q4 and Q8. These two functions explore the impact of haptic feedback and task parameters on learning. As previously mentioned, intermediates may possess a greater amount of practical experience in comparison to novices when it comes to actual surgery. The scores for questions such as LapTool dexterity (Q1), device workspace (Q5), VR and device scaling factor (Q6), task assistance (Q7), and manipulation (Q9) were lower for the intermediate participants compared to the experts. The scores that intermediate subjects gave were higher than those that expert subjects gave for a number of questions. These include how realistic haptic feedback is (Q3) and how it affects learning (Q4), how task parameters affect learning (Q8), electrocauterization (Q11), cloth cutting (Q12), and soft body interactions (Q13). The intermediate and expert scores were nearly equal for the realism of object picking (Q2) and clipping (Q10). Basic operations like clipping and picking show no discernible difference between intermediates and experts. When it comes to complex tasks, intermediates tend to perceive them as more realistic compared to experts. One potential differentiating factor could be the level of realism in haptic feedback during intricate tasks. The expert could assess the complex tasks, including their haptic rendering. However, intermediates lack haptic skills (Prasad et al., 2016), which leads them to perceive complex tasks as more realistic. In other words, intermediates compared the realism of complex tasks using only visual feedback. This can be confirmed by the intermediate score for the question on haptic realism, which surpasses that of the expert. Intermediates found basic LapTool functions more challenging compared to experts, possibly due to their slower adaptation to the simulator. Furthermore, another factor that could contribute to this is the interpretation of task difficulty, which may differ compared to experts. The task difficulty of the intermediates is primarily attributed to controlled force exertion. However, intermediates may have mistakenly attributed the perceived difficulty of the task during manipulation to LapTool’s basic functionalities, such as dexterity and scaling, rather than the realism of haptic feedback. The same can be seen in the lower intermediate score on manipulation compared to the expert. However, further construct validation is necessary to determine the exact reasons for the difference in perception between experts and intermediates and to identify the specific skills that distinguish their experience levels other than haptic perception. Nevertheless, despite the face and content validation, it is intriguing to notice the differences between experts and intermediates. Moreover, the majority of LapTool functionalities have overall scores of four or higher across all subject groups, with the exception of manipulation and electrocautrization, which necessitate additional enhancements. In general, participants with varying levels of experience find the simulator’s functions acceptable.

Novices exhibit a lower standard error than experts and intermediates in both face and content validity. This is primarily due to the sample size considered across the groups. The novice group has a larger sample size (n = 22) compared to the expert (n = 5) and intermediate (n = 5) groups, which suggests that the standard error may be lower. Both the expert and intermediate groups have the same sample size, resulting in nearly identical standard errors.

### 3.2 LapTool tip force model—psychophysical study

**FIGURE 11:**
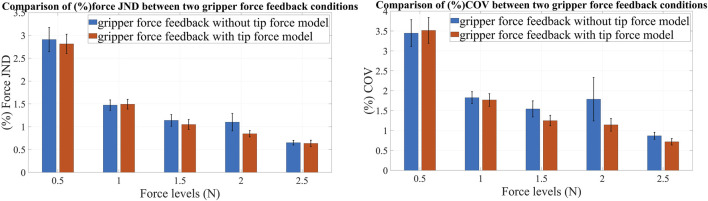
Comparison of JND and COV of force between two conditions of the experiment for different force values; Left: Comparison of (%) force JND for with and without the LapTool tip force model; Right: Comparison of (%) COV of gripping force for with and without the LapTool tip force model. The blue bars show values for the without-tip force model condition, and the orange bars show values for the with-tip force model condition.

The psychophysical study shows that (%) JND and (%) COV are slightly lower at all force levels except for 1 N and 2.5 N, while using the LapTool force model. The error and magnitude of (%) JND and (%) COV decrease exponentially as the force magnitude increases. The (%) absolute force JND and (%) COV are computed for each reference force value using the equations provided in eight and 9, respectively. The (%) JND and (%) COV of force values are compared between two conditions of the experiment: one with and one without the LapTool tip force model for gripper force feedback. The comparison of these parameters shows the difference in user perception between two force feedback methods. We averaged the applied force values over time, determining the (%) absolute JND and the (%) COV of force for each trial and each reference force value. The calculated parameters are subsequently averaged for each reference force value across multiple trials and participants. The mean and standard error of the (%) absolute JND and the (%) COV of force were computed for each reference force value and plotted in Figure 11. The results show that using the force feedback method with the tip force model slightly lowers the (%) force JND compared to force feedback without that. This difference is observed at force levels of 0.5N, 1.5N, and 2N, while there is no discernible difference at force levels of 1N and 2.5N. The decreased (%) force JND for the tip force model in the griping task indicates that including the LapTool tip force model for gripper force feedback improves the user force perception, though the reduction is small. Given that the experiment involves a gripping task, the force JND includes both force control and sensation. As a result, the user has better force control and perception when using the tip force model compared to the direct linear force model. Furthermore, it is evident that the standard error is slightly lower for the tip force model condition at the force levels of 0.5 N, 1.5 N, and 2 N. This shows that the accuracy of force control is better with the addition of a LapTool tip force model in the gripping task.

In addition, for all reference forces other than 0.5 N, the (%) COV of force is lower for the tip force model condition than for the without tip force condition, as shown in Figure 11. This demonstrates that including the tip force model results in a reduction in force variability compared to the direct linear force model. This also suggests that the user has a higher accuracy in controlling force when using the gripper force feedback based on the tip force model. The standard error is lower in the tip force model condition compared to the condition without it at force levels other than 0.5 N. The user performs with less force deviation when using the tip force model. Furthermore, the standard error is high at a low reference force of 0.5 N for both (%) JND and (%) COV of force. This could be due to the difficulty of controlling the applied force at low force levels. The overall average (%) absolute JND for force feedback without the tip force model is 1.45%, while for force feedback with the LapTool tip force model, it is 1.36%. The difference amounts to about 0.1% (0.09). Similarly, the overall average (%) COV of force for force feedback without the tip force model is 1.89%, whereas with the tip force model it is 1.67%. The difference between the two conditions is 0.2% (0.22). The average (%) JND and (%) COV are slightly lower in the tip force model condition, indicating that the user has improved force perception and control. Nevertheless, given the slight differences in the study parameters between the two distinct experimental conditions, it is imperative to assess their statistical significance.

We conducted a one-way ANOVA to determine the significant disparity in parameters between two distinct force feedback methods across the reference force levels. Table 4 provides the ANOVA results for the (%) absolute JND and (%) COV of force for each reference force value. The *p*-value for both the absolute (%) JND and (%) COV of the gripping force is consistently greater than 0.2 (*p* > 0.05) for all reference forces. When averaged over force levels, the *p*-value is 0.251 for the (%) COV of the gripper force and 0.427 for the (%) force JND. Therefore, within the specified force ranges of 0.5–2.5 N, there is no significant difference in study parameters between the two distinct gripper force feedback methods. The results indicate that, though there is a slight decrease in the (%) JND and (%) COV for the tip force model condition across most of the examined force levels, the difference is not statistically significant when viewed across a large group. While the tip force model yields improved user force perception in the current experiment with a small sample size, the differences observed do not significantly vary when considering the entire population. We can infer that the tip force model has minimal impact on the user’s perception of force. Thus, the user perception remained nearly unchanged in both of the examined force feedback conditions.

**TABLE 4:** ANOVA results: (%)JND and (%)COV of gripping force.

Force level (N)	Significance value (p < 0.05)	
	(%)JND	(%)COV
0.5	0.781	0.885
1.0	0.898	0.793
1.5	0.599	0.232
2.0	0.223	0.269
2.5	0.857	0.227

## 4 Discussion

### 4.1 Haptic FLS

Although numerous laparoscopic virtual reality (VR) simulators exist in academic and industrial settings, most of them are primarily focused on enhancing hand-eye coordination. Only a limited number of these simulators are capable of offering force feedback during user interaction. The majority of laparoscopic training programs now adhere to the Fundamentals of Laparoscopic Surgery (FLS) curriculum as the established norm. The FLS tasks highlight precise motion control and dexterity rather than precise force exertion. Even the most important metrics in FLS are time and motion accuracy. Neither the assessment nor the interactions involve any direct force metrics. Furthermore, the existing simulators do not have standard part tasks that specifically focus on force exertion and manipulation. Nevertheless, previous research (Prasad et al., 2016) has demonstrated that the interaction force is a crucial factor that distinguishes the proficiency levels of laparoscopic surgeons, ranging from experts to intermediates and novices. Therefore, it is imperative to incorporate this into the training curriculum. Hence, the developed laparoscopic simulator in this paper proposes a set of part tasks whose design highlights controlled force exertion and manipulation through force feedback. The simulator not only considers the time and accuracy of motion as criteria for completing the task, but it also considers the applied force during different object interactions. The simulator allows the trainer to modify the force feedback, enabling customization of the proficiency level of force exertion for each task and interaction as required. This may help the novice trainees achieve expertise in all aspects of laparoscopic surgery, including haptic skills.

### 4.2 Haptic FLS—face and content validation

The average Likert score for all tasks among participants is approximately 4.6 or higher for face questionnaires and 4.3 or higher for content questionnaires. The observed value is comparable to that of several of the current simulators and aligns with “agree” on the Likert scale. According to the study in (Leijte et al., 2019), the simulator eoSim scored 3.9, 3.6, and 3.7 for realism, 4.0, 3.4, and 3.7 for didactic value, and 4.2, 3.7, and 4.0 for usability across three distinct suturing tasks. The expert’s score was marginally lower than the novice’s for two of the needle transfer tasks, but there was no difference in their scores for the suturing. Nevertheless, the disparity did not show statistical significance. As shown in the (Xiao et al., 2014) study, the Ergo-Lap Simulator has a face validity score of almost four for tasks that involve moving beads, tubes, stretching bands, and suturing. Nevertheless, there is no specific analysis for the face validity comparison between experts and novices. The VR simulator named SINERGIA has been validated through face validity for tasks related to coordination, navigation, and grasping (Sánchez-Peralta et al., 2010). The questionnaire covered a wide range of topics, including realism, haptic experiences, and the utilization of the simulator. The observed score for novices ranged from 4.14 to 3.07, whereas for experts, it varied from four to 2.83. The usability aspect received the highest score, reaching nearly 4, while the haptic sensation aspect acquired the lowest score, roughly 3. Additionally, there was a slight increase in the score of novices compared to experts, although the difference is not statistically significant.

The study conducted in (Schreuder et al., 2009) investigates the face validation of the Laps VR simulator developed by Surgical Science. The study is mostly about testing the simulator on four basic surgical skills and three specific procedure modules. These include tubal occlusion, salpingectomy in ectopic pregnancy, and the last stage of suturing in a myomectomy. The average score for realism ranged from 2.43 (suturing) to 3.94 (appearance of the Laptools), while for training capacity, it varied from 3.13 (myoma suturing) to 4.46 (hand-eye coordination). The questions on tissue reaction for manipulation received the lowest scores, with a mean rating of 2.79. Similarly, the appearance of needle and thread and myoma suturing questions had mean ratings of 2.87 and 2.43, respectively. The expert’s score was slightly lower than the novice’s in certain surgical abilities, but there was no disparity in LapTool dexterity. The study in (Escamirosa et al., 2015) examines the face and content validity of the EndoViS training simulator. The validated tasks include peg transfer, rubber banding, pattern cutting, and intracorporeal suturing. The questions encompassed aspects of the simulator’s design, realism, and functionality. The trainer’s overall Likert score was 4.18, with realism receiving the lowest value. Experts scored lower than both intermediates and novices, with a statistically significant difference only observed in the case of laparoscopic tool movement. As stated in the research under discussion, a score above four in the current validation indicates that the developed simulator is generally accepted for its fundamental appearance and functionality. Additionally, the questions “Did the parameters help to improve the learning curve?” and “Did the presence of haptic feedback improve the learning experience?” received scores higher than four on average. This indicates that the developed simulator has provisions for force skills training and assessment.

The average scores for each task question ranged between four and five in both face and content validity. The minimum score of three indicates areas that require improvement. Face validity showed that it is necessary to improve the realism of the supporting components and the soft body. In terms of content validation, the lapTool manipulation received a low score. We observed only a few outliers on the face validity, indicating the realism of the supporting parts. There were a few outliers in the content validity, which belong to novices who find the bi-manual tasks difficult. The minimal score and outliers in the box plot indicate that there is bias, possibly due to novice subjects’ perceptions of task difficulty or specific simulator limitations. Therefore, we further analyzed the results based on the subject’s surgical expertise to pinpoint the simulator’s limitations. We conducted a comparative analysis of the responses provided by experts, intermediates, and novices on a range of tasks and common questions. For most of the questions, the scores of experts and intermediates were generally lower than those of novices. While the disparity in scores between subject groups is only significant for a few questions, the trend is nevertheless evident in the majority of the questions. As mentioned in Section 3.1, the disparity in subject scores may be attributed to the reference system used for comparison. Experts may have evaluated the simulator by comparing it to actual surgery, whereas novices may have assessed it only based on the box trainer and surgical videos. Put simply, the simulator has the potential to be similarly realistic as the box trainer, but it requires more enhancements to be suitable for real surgical procedural training.

Only the intricate tasks demonstrated the disparity between experts and novices, while the fundamental tasks and functions showed no distinction in terms of both face and content validity. In terms of content validity, there were no discernible differences in the scores between experts and novices when it came to fundamental LapTool functions. However, we observed differences in more complex surgical tasks like cutting, clipping, and soft body manipulation. These findings reinforce the idea that experts and novices use different reference systems when comparing the simulator functions for these challenging tasks. Hence, the basic LapTool functions in the simulator are realistic, as are box trainers and real surgical LapTools; however, they require enhancement in advanced surgical interactions. Similarly, the distinction between intermediates and experts is only evident in the context of LapTool functions, while the ratings for surgical interactions were nearly identical. This may be attributed to the misconception of perceived level of task difficulty by intermediates, which is actually related to the haptic skills as stated in Section 3.1. Analyzing the expert scores revealed the need to improve the following functions of the simulator: twisting movement, haptic feedback, and electrocautrization sparks. Furthermore, the simulator demonstrates dexterity comparable to real surgical laparoscopic tools. The average score for both face and content validity across all subject groups exceeds four for all tasks and questions, indicating that participants with varying levels of surgical experience accept the simulator.

The average score for the realism of haptic feedback ranges from 3.95 to 4.43 across different surgical groups. The observed validation score is comparatively greater than the scores observed for haptic feedback on the previously discussed existing simulators. The score represents the “agree” response on the Likert scale, indicating acceptance of the simulated haptic feedback and haptic interactions. This suggests that the HFLS possesses better haptic simulation. Nevertheless, it is not feasible to directly compare HFLS with any of the current simulators due to variations in the experimental setup among them. Moreover, performing additional validations, specifically construct validity, on the HFLS tasks and metrics will provide further confirmation of its suitability for use in VR simulator training. The mean score for task parameters ranges from 4.45 to 4.65 among various subject groups. This shows the acceptability and impact of proposed measures on VR laparoscopic training.

### 4.3 LapTool force model—force JND experiment

In the force JND experiment, it was found that the (%) force JND and (%) COV are a little lower when the LapTool tip force model is used for gripper force feedback than when it is not. However, the difference between experimental conditions was only 0.1% and 0.2% for (%) force JND and (%) COV of force, respectively. These differences do not show statistical significance. Therefore, we can conclude that the inclusion of the LapTool tip force model in gripper force feedback did not improve the user’s perception and control of force. Incorporating a lapTool force model enhances the accuracy of force feedback. Nevertheless, the inclusion of this model does not alter the user’s perception of the experimental force range, which spans from 0.5 to 2.5 N. This could be due to the dominance of visual feedback in the VR simulation. When the visual feedback is more dominant, even simple linear force feedback is enough to create a realistic perception (Hyun et al., 2004). The visual feedback could add a psychophysical filter to the user’s force perception. Further research will elucidate the impact of multi-modal stimuli on user perception. Additionally, the type of visual force feedback utilized in the experiment could be another factor. The tip force is used to give visual feedback on the applied force in both experimental conditions, though the force rendered to the user differed. This further supports the notion that the visual force feedback dominates the perceived force feedback. Hence, further experiments without visual force feedback or with visual force feedback of the handle force may provide more explanation.

The observed (%) JND of 1.45% and 1.36% is lower than the (%) JND seen in previous studies (Prasad et al., 2014). The force JND observed was 5.14% for the dominant hand while using the laparoscopic tool. However, the previous study considered a contra-lateral force matching paradigm and a probing task that involved the entire movement of Laptool. In the present study, only grasping is considered, without any additional LapTool movements in other directions. Other possible causes for the observed lower JND value could be that the task involves the whole hand and a static VR object. Apart from the above study, there are not many studies available in the literature with the same experimental setup for (%) force JND comparison. Moreover, the considered force range of 0.5–2.5N for an experiment was low compared to the actual operating force range (0–10N) of laparoscopic surgery (Yamanaka et al., 2015). Hence, it is necessary to study the effects of the lapTool force model at higher force ranges to conclude its applicability. The current study suggests that a simple linear model, rather than a complex non-linear force model, suffices to render the gripper force feedback in the VR simulation. However, further studies with multi-model stimuli, a higher range of forces, and subjects with varying laparoscopic experiences will show the usability of the force model for gripper force rendering.

## 5 Conclusion

To emphasize haptic skill training in VR laparoscopic training simulators, we proposed haptic-based FLS (HFLS) part tasks. The HFLS tasks are simulated in VR and interfaced with a customized five-DOF bi-manual haptic device for force feedback. Each HFLS task focused on the different skills required for laparoscopic surgery. These tasks highlight the controlled force exertion and simulate the interactions accordingly. The trainer can customize the threshold force value for specific skills and assessment metrics. Using simple linear force models, we calculated the reaction force for various object interactions. Different parameters, such as time, LapTool velocity, force, and other error metrics, were measured and displayed in real time. The Haptic-VR plugin was developed to interface the haptic device and library with VR simulation. Face and content validations were conducted to validate the function and realism of the simulation. The validation results showed that subjects with different laparoscopic experiences accepted the simulation. We also implemented the LapTool force model in the simulation to enhance the realism of gripper force feedback. We conducted a psycho-physical force JND experiment for the laparoscopic gripping task to study the enhancement in user force perception due to the force model. The results showed no significant difference in user force perception. However, the force model improves the accuracy of object interaction during the gripping task. Hence, a linear force model could be sufficient for realistic gripper force rendering in the force range of 0.5–2.5 N.

### 5.1 Limitations

Face and content validation have the limitation of including fewer expert subjects than novices. The current study collects the subject’s surgical experience in terms of years of experience, not the number of surgeries. The face and content validity questionnaire does not include the simulator’s assessment because the assessment parameters have to be studied through construct validity. Moreover, the HFLS does not simulate the suturing task, necessitating its inclusion to ensure comprehensive training in basic skills. For the force JND experiment on gripper force feedback with the LapTool force model, only the static cube is considered to avoid any dynamic effect on force rendering. Furthermore, the subjects in the study are novices with no prior laparoscopic surgical experience.

### 5.2 Future works

Further construct validity is required to demonstrate the simulator’s effectiveness in differentiating the subjects of different laparoscopic experiences, as well as the possibility of including it in training. For the laparoscopic tip force model, further studies with higher force range values are required to explain the actual use of it in the simulation. Furthermore, conducting studies with dynamic VR objects could uncover any interdependencies between the object and the tip force model. A force JND study with laparoscopic surgeons will show the relationship between the tip force model and expert force perception. Furthermore, the current study solely presents the reaction force as a visual indicator of the applied force. Measuring the actual force applied by the user will help in understanding the force JND for real and simulated force feedback.

## Data Availability

The raw data supporting the conclusion of this article will be made available by the authors, without undue reservation.
